# Ignoring a noisy metronome during dyadic drumming

**DOI:** 10.3389/fpsyg.2026.1929677

**Published:** 2026-09-30

**Authors:** Laura Bishop, Dongho Kwak

**Affiliations:** 1 RITMO Centre for Interdisciplinary Studies in Rhythm, Time and Motion, University of Oslo, Oslo, Norway; 2 Department of Musicology, University of Oslo, Oslo, Norway

**Keywords:** attention, body motion, coordination, expertise, joint action, sensorimotor synchronization

## Abstract

**Introduction:**

This study investigated how the tonal and rhythmic properties of a “noisy metronome” and the opportunity for visual contact would affect drumming partners' abilities to synchronize with each other.

**Method:**

Participants' task was to drum a regular beat in synchrony with their partner at a prescribed tempo. They heard a metronome while drumming that they were instructed to ignore. We manipulated the noise profile of the metronome so that it contained long-range autocorrelations, short-range autocorrelations, or white noise. We also manipulated its pitch so that it formed a consonant or dissonant chord alongside the participants' drum tones. We assessed the effect of this noisy metronome on participants' synchronization stability, tempo, and body motion.

**Results:**

Our findings showed that the noisy metronome impaired synchronization between partners but improved tempo-keeping. Manipulations of the metronome's rhythmic and tonal properties had no effect on any of the outcome variables. The noisy metronome prompted increased head and hand motion from the participants (measured in terms of motion speed); head motion was also greater among active musicians than inactive/nonmusicians, and hand motion was greater among partners who knew each other. Visual contact improved synchronization and participants moved their drumming hand more when they could see each other than when they could not.

**Discussion:**

Our findings have implications for our understanding of how people process and respond to competing rhythms and the strategies they use to (co-)regulate the timing of their movements.

## 1 Introduction

In our daily lives, we are surrounded by rhythms—some that we produce ourselves and others that we perceive from external sources. Sometimes, we are faced with the task of synchronizing a rhythm that we produce with a rhythm that we perceive. This is commonly the intention of people playing music or dancing together and is also important for sports like rowing. In interactive settings, people adapt to and shape one another's rhythms in real-time. This contrasts the unidirectional information flow that occurs when, for example, a musician practices with a metronome.

When presented with an external periodic rhythm, a person may entrain to it, meaning that the rhythm exerts an influence on the person's internal time-keeping (Clayton et al., 2020). Entrainment can occur bidirectionally if the source of the external rhythm is also susceptible to influence. To synchronize their actions with an external rhythm, people engage a combination of anticipatory and reactive cognitive mechanisms (Keller et al., 2014). Anticipation involves estimating the timing of future events in the rhythmic sequence based on the timing of previous events. Reactive error-correction operates via two processes. Phase correction, which is automatic, compensates for the amount of leading/lagging between events with adjustments to how internally-generated pulses align with externally-generated pulses. Period correction, which requires attention (van der Steen and Keller, 2013), involves adjusting the period of an internal timekeeper to match the intervals generated by an external timekeeper. Together, these processes allow people to cope with temporal variability in their own and the external rhythm.

The current study is concerned with what happens when a person perceives two non-synchronous rhythms and tries to synchronize their own drumming actions with one of them. Such a task requires the person to segregate the rhythms perceptually into separate streams, then align their actions with one (target) stream while resisting influence by the other (distractor) stream. Several studies have examined versions of this task, manipulating the temporal relationship between target and distractor (Repp, 2003, 2004), whether they are presented in the same or different modality (Repp and Penel, 2004), or whether they constitute speech or music (Dalla Bella et al., 2013; Białuńska and Dalla Bella, 2017).

The general finding is that the distractor rhythm tends to disrupt synchronization with the target, “attracting” participants' actions (which usually comprise tapping). The studies cited above have considered possible theoretical explanations for why this disruption occurs. One possibility is that perceptual integration occurs for target and distractor tones that fall close together in time, triggering automatic phase-correction mechanisms that adjust actions in the direction of the distractor. Repp's (2004) finding that the extent of disruption depended on absolute temporal separation rather than the relative phase between target and distractor tones was in line with this hypothesis.

Another (not incompatible) possibility, based on a dynamical systems perspective (see Large and Jones, 1999), is that internal oscillators entrain to both target and distractor rhythms independently, and the rhythms compete with one another for attention. Rhythms having a metrical structure, as found in music, trigger entrainment particularly strongly (Nozaradan et al., 2011), which might be why distractors comprising (metrical) music are more disruptive than distractors comprising non-metrical speech. This difference between musical and spoken distractors disappears when the spoken distractor comprises spoken lyrics presented with isochrony and a metrical structure (Dalla Bella et al., 2013).

The current study adds to this discussion with an examination of how distractor rhythms' timing and pitch attributes affect participants' synchronization with target rhythms. In terms of timing attributes, the question we asked was whether distractors' predictability would affect how strongly they attracted participants' actions. People learn slowly-unfolding statistical patterns in the rhythms they perceive, which may influence attention during perception and synchronization (Stephen et al., 2008). Of particular interest across a number of domains are rhythms that exhibit long-range autocorrelation (LRC), a statistical feature of time series data with a long-lasting memory (Tanaka-Ishii, 2018), meaning that past events persistently impact future events over time. Rhythms with LRC introduce a level of unpredictability with an asymptotic power-law decay in autocorrelation analysis. This implies that when analyzing the correlation between time intervals in these sequences, the decay pattern follows a power law, indicating a slow and persistent decrease in correlation over time (Torre et al., 2013; Tanaka-Ishii, 2021, p. 27). Rhythms with LRC are found in complex systems consisting of many smaller parts, including natural, biological, and physical systems (Sornette, 2004; Delignières and Marmelat, 2013; Bassingthwaighte et al., 2013). Timing fluctuations in human musical performances show LRC characteristics, and listeners prefer such variations over randomized (white noise) variations (Hennig et al., 2011). We tested the hypothesis that distractor rhythms containing LRC would be more predictable than those containing short-range correlations (SRC) or white noise (WN), and therefore compete more strongly with target rhythms for attention.

In terms of pitch attributes, the question we asked was whether the pitch relationship between the distractor and participants' own drumming sounds would affect the distractor's influence on participants' drumstrikes. Pitch relationships are a defining feature of many rhythms and form a basis for musical harmony. Combinations of pitches can be described as consonant (perceived as smooth and stable) or dissonant (perceived as rough and unstable; Di Stefano et al., 2022). In Western music traditions, consonance and dissonance are linked to emotional valence and can be used to create feelings of tension and relaxation. They can affect synchronization accuracy, with more accurate and stable synchronization observed for consonant sounds than for dissonant sounds (Komeilipoor et al., 2015). Outside of a rhythmic context, perception of consonant and dissonant intervals evoke attention differently, with consonant intervals prompting slower behavioral responses and stronger neural markers of motor inhibition and attention engagement (Sarasso et al., 2019). Given these findings, we tested the hypothesis that consonant intervals between distractor tones and participants' drumming tones would be more attention-grabbing than dissonant intervals.

In addition to introducing these manipulations of distractor timing and pitch attributes, the current study adapted the experimental paradigm into a real-time dyadic synchronization task. Therefore, instead of synchronizing with a disembodied, non-adaptive sound signal, participants synchronized with each other. This enabled us to investigate the interactive, embodied strategies that people use to resist distractor rhythms' influence.

When synchronizing rhythmic actions, people commonly draw on other bodily modalities to help regulate their timing. Visual cues can activate internal simulation of others' actions, facilitating prediction of action effects (Calvo-Merino et al., 2006). Studies employing joint action tasks, such as synchronizing reaching movements or building an object together, show that people co-represent aspects of a partner's task, adapting their motion to accommodate their partner, even if they are not instructed to do so (Moreau et al., 2016; Schmitz et al., 2017).

In real-world musical settings, people who are playing together can often see (and sometimes kinaesthetically feel) each other's movements. Musicians have been shown to modulate their sound-producing and ancillary (i.e., not directly sound-producing) motion in ways that make it more communicative; for example, making larger-amplitude movements and/or moving more predictably (Glowinski et al., 2013). Musicians also change the way they move to regulate their own performance, for example, by moving rhythmically (Laroche et al., 2022) or more forcefully (Keller et al., 2010) to maintain accurate timing. These changes support rhythmic accuracy and coordination, and are part of the broader landscape of gestural communication that plays out during musical interaction, which also includes socially-significant gestures, such as smiling, and responsive or imitative behaviors that communicate approval and engagement (Davidson, 2012; Duranti et al., 2021).

A number of studies have tested whether visual contact improves synchronization between musical partners (Kawase, 2014; Bishop and Goebl, 2015; D'Amario et al., 2018; Colley et al., 2018; Bishop et al., 2019; Palmer et al., 2019). The results are mixed and suggest that the benefits of visual contact depend on the specific demands of the task, including the complexity of the actions to be synchronized and the features of the motion cues that are given. A common finding is that visual cues tend to facilitate synchronization when a rhythm's timing is difficult to predict, for example, when there are tempo changes (Kawase, 2014; Colley et al., 2018), pauses (Bishop and Goebl, 2015), or the absence of a regular beat (Bishop et al., 2019). Outside of these cases, visual cues tend to be unnecessary as people are able to rely on (largely automatic) error correction mechanisms. In the context of the current study, we tested two hypotheses related to visual communication: First, that rhythmic distraction would prompt participants to move more to help regulate their own timing and communicate with their partner; and second, that visual cues would help participants perceptually segregate distractor and partner rhythms and maintain attention on their partner's beats, improving synchronization stability.

The experimental paradigm we used draws on processes that musicians might be expected to have strengthened through practice. Prior studies have shown that musicians outperform non-musicians on tasks evaluating—among other processes—auditory stream segregation (Parbery-Clark et al., 2009), auditory attention (Strait et al., 2010), musical, verbal, and visuospatial short-term memory (Grassi et al., 2025), pitch discrimination (Tervaniemi et al., 2005), beat perception, sensorimotor synchronization, and synchronization-continuation (i.e., synchronizing with a metronome, then maintaining the same tempo through a period without metronome; Matthews et al., 2016). Musicians might also have learned deliberate strategies for keeping time while performing and coordinating their actions with others, for example, making use of breathing or ancillary movements. We included a wide range of musical backgrounds in our participant sample and treated “musical activity” (i.e., whether a participant was currently engaged in regular instrumental practice) as a covariate in our analyses.

Our study addressed three primary research questions: First, how does the presence of a variable distractor rhythm (“noisy metronome”) affect the synchronization stability between two people who are drumming together? Second, how do timing and pitch features of the distractor rhythm modulate these effects? Third, how do people use visual-motor cues to support synchronization with their partner in the presence of a distractor rhythm? Participants drummed together in pairs and completed a synchronization-continuation task. Their instructions were to synchronize as closely as possible while maintaining a prescribed tempo and ignoring the noisy metronome. It was important that we included an instruction to maintain the prescribed tempo so that participants would not simply adopt a different tempo as a way of avoiding interference from the noisy metronome. We manipulated as independent variables (i) the type of variability in the noisy metronome (LRC, SRC, WN), (ii) the pitch of the tone produced by the noisy metronome (forming a consonant or dissonant chord with the participants' tones), and (iii) whether or not the participants could see each other while drumming. A baseline trial without the noisy metronome was also captured for each dyad.

We tested four hypotheses:

1. Distractor rhythms containing LRC will be more predictable than those containing short-range correlations (SRC) or white noise (WN), and will have a more destabilizing effect on partner synchronization.
2. Consonant intervals between distractor tones and participants' drumming tones will attract attention more strongly, and have a more destabilizing effect on partner synchronization than dissonant intervals.
3. Rhythmic distraction will prompt participants to exaggerate their body motion to regulate their own timing and communicate with their partner, and as a result, the presence of the distractor rhythm and visual contact will increase body motion.
4. Visual cues will help participants segregate distractor and partner rhythms and maintain attention on their partner's beats, improving synchronization stability.

The study had five dependent variables. *Synchronization precision* was our primary index of synchronization stability within dyads. *Distractor influence* was additionally computed to compare the correspondence in drumming timing between partners against the correspondence between either partner and the distractor. *Tempo accuracy* was calculated to confirm whether participants carried out the task as instructed. Finally, we computed *quantity of head motion* and *quantity of vertical hand motion*—measured in terms of motion speed—as indications of how participants adapted their communicative and self-regulatory motion to the task conditions.

## 2 Materials and methods

### 2.1 Participants

An *a priori* power analysis for repeated-measures ANOVA showed that a sample size of 38 participants was needed to detect a small effect size (*d* = 0.2) with 95% power at an alpha of 0.05. A total of 50 adult participants completed the experiment (26 women, 23 men, and 1 non-binary), for a total of 25 dyads. They reported an average age of 26.8 years (*SD* = 6.9). Twenty-three were active musicians with an average of 8.4 years regular practice (*SD* = 6.5), 11 were inactive musicians who were no longer playing, but had an average of 4.4 years regular practice in the past (*SD* = 2.5), and 16 were non-musicians with less than two years experience on any instrument. The most common currently-played instruments were guitar, piano, bass, and drums, and the most common previously-played instruments were drums, piano, guitar, saxophone, clarinet, and bass. Participants were paired according to their availability and were permitted to register with a friend; musical background was not taken into account. In the end, there were seven dyads with two active musicians, nine dyads with no active musicians, and nine dyads that were mixed in musical background. Participants reported that they knew each other in 15 of the 25 total dyads. One dyad was excluded from the analysis because they did not follow the instructions properly.

### 2.2 Ethics

The study was conducted in line with international standards for research involving human participants. Participants provided written informed consent and received a gift card as compensation.

### 2.3 Design

We used a within-subjects design and tested three independent variables that were central to our hypotheses (2 × 3 × 2): visual contact (yes or no), type of distractor noise [LRC, SRC, or white noise (WN)], and distractor pitch (perfect 5th or diminished 5th). We additionally included two between-subject variables as covariates in our analysis: participant musical activity (active musicians or inactive/nonmusicians) and partner familiarity (partners knew each other or not). These variables were not part of our original design, but emerged in our participant sample, so we included them in our analyses to test whether they could account for some variability in the data.

Overall, there were 12 experimental trials, each presenting a different condition, plus one baseline with no distractor. Trials were presented in a randomized order for each dyad, apart from the baseline, which was always completed first. The baseline also served as practice to ensure that participants understood the task. We had five dependent variables: synchronization precision, distractor influence, tempo accuracy, quantity of head motion, and quantity of hand motion. These are defined in the Analysis section.

### 2.4 Rhythm stimuli

Timestamps for the distractor rhythms were generated in advance using the “fracdiff.sim” function from the *fracdiff* library in R. For rhythms with LRC, SRC, and white noise, respectively, the autoregressive parameter was set to 0, 0.6, and 0, and the fractional differencing parameter was set to 0.45, 0, and 0. Simulated data for each rhythm were rescaled to have a mean inter-event interval of 800 ms and a range of 680–920 ms. We chose an inter-event interval of 800 ms so the tempo would be slower than the average spontaneous motor tempo of most adults, making it more challenging to maintain. Example stimuli for each type of noise are shown in Figure 1.

**Figure 1:**
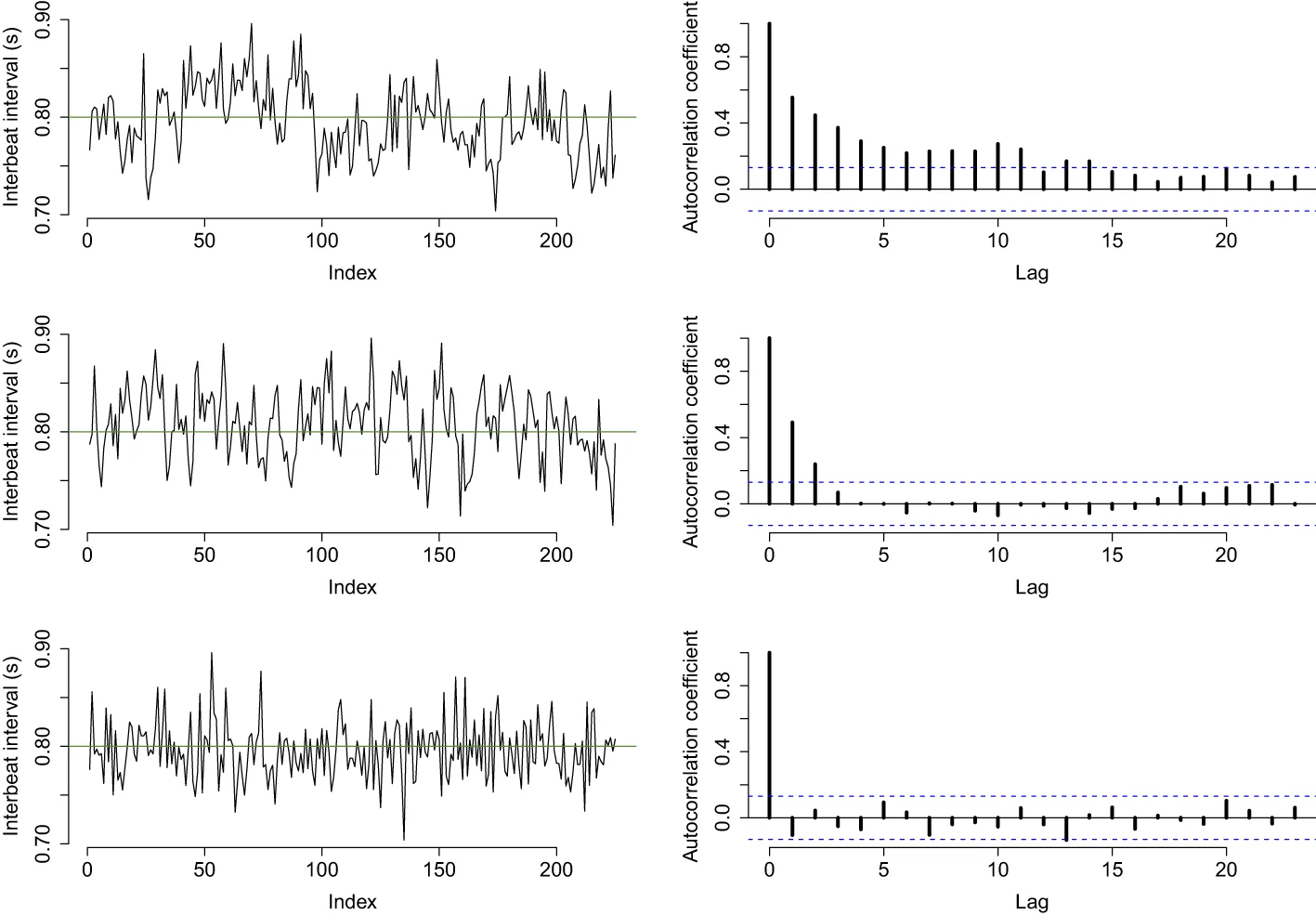
Example distractor rhythms with LRC **(top)**, SRC **(middle)**, and WN **(bottom)**. Plots on the left show the series of interbeat intervals, with a green horizontal line indicating the mean (0.8 s). Plots on the right show the series of autocorrelations per lag for each rhythm, with blue horizontal line indicating the confidence intervals (95%).

Participant dyads were randomly assigned to one of three stimulus groups (8–9 dyads per group). For each group, we generated four different stimuli with each type of noise (LRC, SRC, and white noise). This meant that dyads could play with a different stimulus in every trial and never hear the same stimulus twice. The between-subjects manipulation of stimulus group was not meant to address any of our hypotheses, but was included to ensure that our results could not be attributed to the unique characteristics of a specific stimulus set. A circular ANOVA showed that stimulus group had no statistical effect on synchronization precision, *F*_(2, 23)_ = 2.41, *p* = 0.11 (see Analysis for our definition of synchronization precision). Therefore, this manipulation was not included in any further analyses and is not discussed further.

### 2.5 Experiment setup

Participants sat facing each other in the center of our motion capture lab (Figure 2). They each played with a drumstick on a MIDI drum pad (PDX-8 toms from a TD-17 V-Drumset), which was attached to a stand and adjusted to a comfortable height. They wore Beyerdynamic DT 770 Pro headphones, through which they heard their own sound, their partner's sound, distractor rhythms, and a metronome that played eight beats to set the initial tempo. A Qualisys system with 12 Oqus 300 cameras was used to capture motion data. Participants also wore eye-tracking glasses and electromyeography sensors, but those data are not included in the present study.

**Figure 2:**
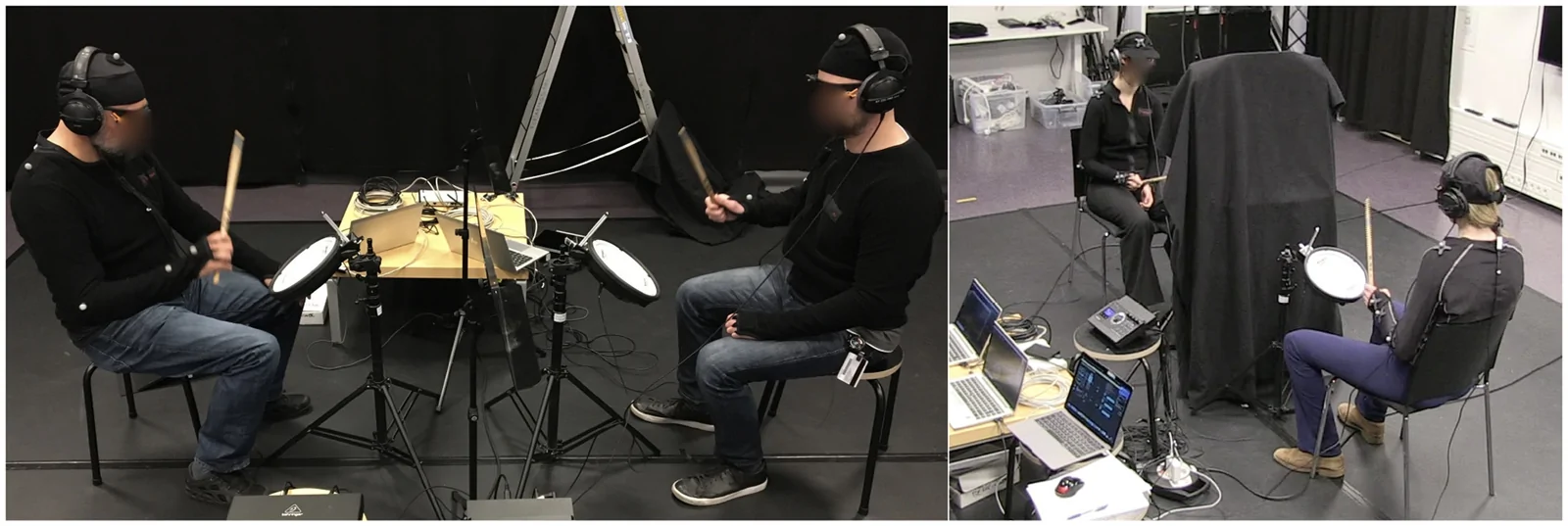
Photos showing the experiment setup, at left, with visual contact between participants, and at right, with a curtain hung to prevent visual contact.

We controlled the experiment and collected MIDI data using a custom patch in MaxMSP. MIDI from the drum pads were sent into the patch, which returned sound through participants' headphones. Participants heard their sound and the noisy metronome as unique xylophone tones. One drum produced a C5, and the other produced an E5. These pitch mappings stayed the same throughout the experiment, so the pitch played by individual participants was always the same. As explained under Design, the noisy metronome played either a G5 or a G♭5.

### 2.6 Procedure

At the start of the session, participants tried out the drum pads and familiarized themselves with the sound that their own and their partner's drumming made (i.e., who played which pitch). They then completed a 30-s resting baseline (for eye-tracking and EMG recording), followed by a 1-min drumming baseline. To initiate the drumming baseline, a cueing-in metronome played eight beats at a tempo of 75 bpm using a click sound. We instructed participants to join in and synchronize with the metronome when they were ready, then continue drumming at the same tempo while synchronizing as accurately as possible with their partner.

Following the baseline, participants completed the 12 experimental trials, each 2 mins long. We explained that they would hear the sound of a third “drummer” throughout the 2-min trials, which they should ignore. Their goal remained the same as in the baseline: join in and synchronize with the introductory metronome, then maintain the same tempo while synchronizing as accurately as possible with their partner. Trials always started with 8 metronome clicks at 75 bpm. Participants were allowed a brief break between trials, and told the experimenters when they were ready to continue. During trials with no visual contact, we hung a curtain between partners so they could not see each other at all (Figure 2). At the end of the session, participants completed a brief demographic questionnaire.

### 2.7 Analysis

In this section, we describe our pre-processing procedures, introduce how each of our dependent variables was calculated, and describe our approach to statistical analysis.

#### 2.7.1 MIDI data pre-processing

MIDI data files often contained extraneous drum strikes outside the official trial start and end times. In addition, participants sometimes drummed inconsistently when first joining in with the metronome. We wanted to exclude these periods of variable drumming from our analysis, so implemented the following procedure. First, we found the 2-min interval in each trial that contained the most drum strikes. We then trimmed 5 s off the start and end of this 2-min interval. Only data within the remaining 110 s were retained for analysis.

For each participant and trial, the timestamps of drum strikes were differenced once to obtain a series of Interbeat Intervals (IBIs). Some trials contained very short IBIs (<200 ms), which arose when the drumstick bounced on the pad immediately following a strike. These “bounces” were discarded. Some trials also contained very long IBIs, which occurred when participants got out of sync and paused before re-establishing synchronization. We removed these outliers for the analysis of Distractor Influence (described below), where they disproportionately affected the results. An upper threshold was set at 1,200 ms (1.5 times the expected IBI of 800 ms), and IBIs that were longer than this threshold were omitted. A linear interpolation was then made to fill the resulting gaps.

#### 2.7.2 Synchronization precision

We used circular statistics to assess the precision of synchronization within dyads. We modeled our analysis after Pecenka and Keller (2011), who tested synchronization in dyads in the absence of a metronome. With circular statistics, asynchronies between participants are measured as angular deviations relative to a reference period. Our participants did not consistently maintain the metronome tempo of 75 bpm, so we could not use this tempo to define our reference period. Instead, we made a rough alignment between participants' drum strikes using a nearest neighbor method (“nn2” function from the *RANN* library in R; Arya et al., 2019). We then computed angular deviations between participants' drum strikes twice, once using the IBIs from one participant as instantaneous reference periods, and then a second time using the IBIs from the other participant. Our dependent variable for synchronization precision was the mean resultant length, a measure that is inversely related to circular variability. This was computed using the “rho.circular” function from the R library *circular* (Agostinelli and Lund, 2023).

#### 2.7.3 Distractor influence

We used cross-correlations to estimate the influence that the noisy metronome had on participants' timing and the influence that participants had on each other's timing. Cross-correlations were computed for the range of –1 to +1 lag and the maximum signed coefficient was extracted per trial and participant. We computed correlations at the individual rather than dyad level because the noisy metronome could influence participants individually. Thus, the coefficient representing participants' influence on each other was the same for both partners in a dyad, but each partner had their own coefficient representing influence from the noisy metronome.

#### 2.7.4 Tempo accuracy

We operationalized tempo accuracy in terms of how much participants drifted from the cueing-in tempo of 75 bpm. Participants' IBIs were combined into a single vector per trial, and a linear model was fit. The model coefficient for the effect of time on IBI was our dependent variable for tempo accuracy.

#### 2.7.5 Quantity of head and vertical hand motion (QoM)

Raw position data for the head and drumming hand were smoothed using a Savitzky-Golay filter (window size = 11; *prospectr* library in R) and velocity was derived. For head motion, the Euclidean norm was then computed. We used the mean of the head velocity norm (i.e., head speed) per trial and participant as our measure of head QoM. For hand motion, we analyzed data only in the vertical axis. We used the mean of absolute vertical velocities (i.e., head speed) per trial and participant as our measure of hand QoM.

#### 2.7.6 Evaluating effects of manipulated variables

We used Linear mixed effects models (LMM) paired with ANOVA to test for relationships between our independent variables (distractor noise, distractor pitch, and visual contact), covariates (participant musical activity and partner familiarity), and our dependent variables (synchronization precision, distractor influence, tempo accuracy, quantity of head motion, quantity of hand motion). LMMs were first constructed using the *lmerTest* library in R, then run through an ANOVA to obtain *F* and *p*-values. Significance was evaluated at α = 0.05, except in the case of *post-hoc* tests, where we used Bonferroni corrections (see details in the relevant Results sections).

For four of our dependent variables (all except distractor influence), our analysis proceeded in two stages: First, we compared distractor conditions against the baseline; next, we ran separate models for each independent variable to test between distractor types, excluding data from the baseline. Distractor influence was not computed for the baseline, since there was no distractor in the baseline. Therefore, we only needed one step, which tested for interactions between distractor variables and comparison (participant vs. metronome or partner vs. partner). All of our models included a random intercept either for dyad ID (for synchronization precision, tempo accuracy, and quantity of motion) or for participant ID (for distractor influence).

## 3 Results

Our results are presented below, organized by dependent variable.

### 3.1 Synchronization precision

We predicted that synchronization precision would be reduced by the noisy metronome, particularly in the LRC, perfect fifth, and no-visual-contact conditions. As predicted, synchronization precision was lower with the noisy metronome than in the baseline, *F*_(1, 23)_ = 5.05, *p* = 0.03, and $\eta_{p}^{2}$ = 0.18; the covariates (participant musical activity and partner familiarity) had no significant effect. There were no differences between distractor noise or pitch conditions and no covariate effects for either model. Synchronization precision was greater when participants could see each other than when they could not, *F*_(1, 23)_ = 13.49, *p* = 0.001, and $\eta_{p}^{2}$ = 0.37, with no significant effect from either covariate. Boxplots for synchronization precision are shown in the top line of Figure 3.

**Figure 3:**
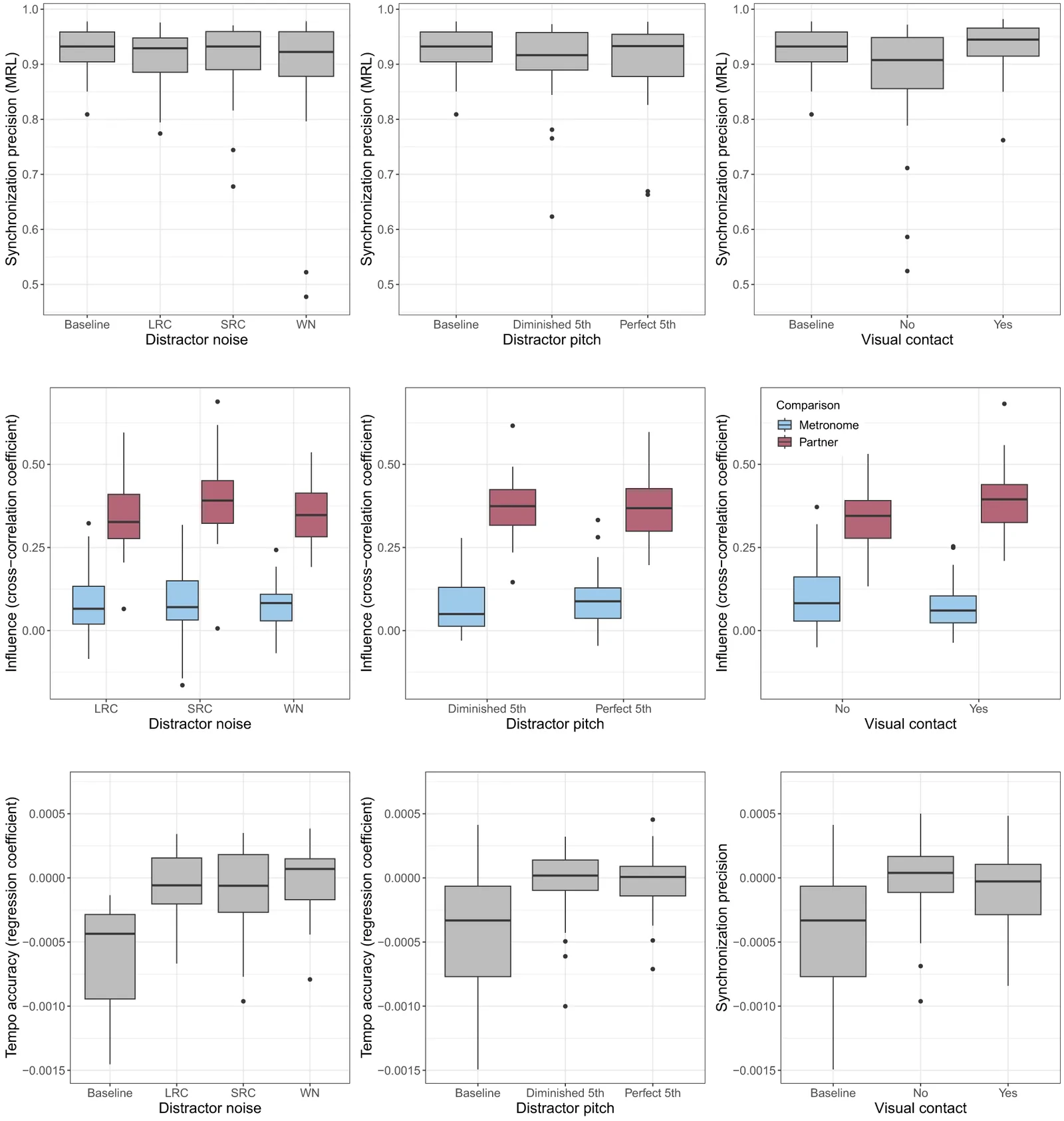
Effects of distractor noise, distractor pitch, and visual contact on synchronization precision **(top)**, distractor influence **(middle)**, also including effects of comparison, and tempo accuracy **(bottom)**. MRL refers to mean resultant length.

Figure 4 shows the distribution of asynchronies in two example baselines and noisy metronome conditions. The dyad whose data are in the top plots synchronized precisely in both trials. The dyad shown in the lower plots was profoundly destabilized by the noisy metronome. For each dyad, three plots are shown: asynchronies between partners during the baseline (left), a noisy metronome condition (center), and asynchronies between participants and the metronome during the same noisy metronome condition (right). For both dyads, noisy metronome plots come from perfect 5th-WN trials with visual contact.

**Figure 4:**
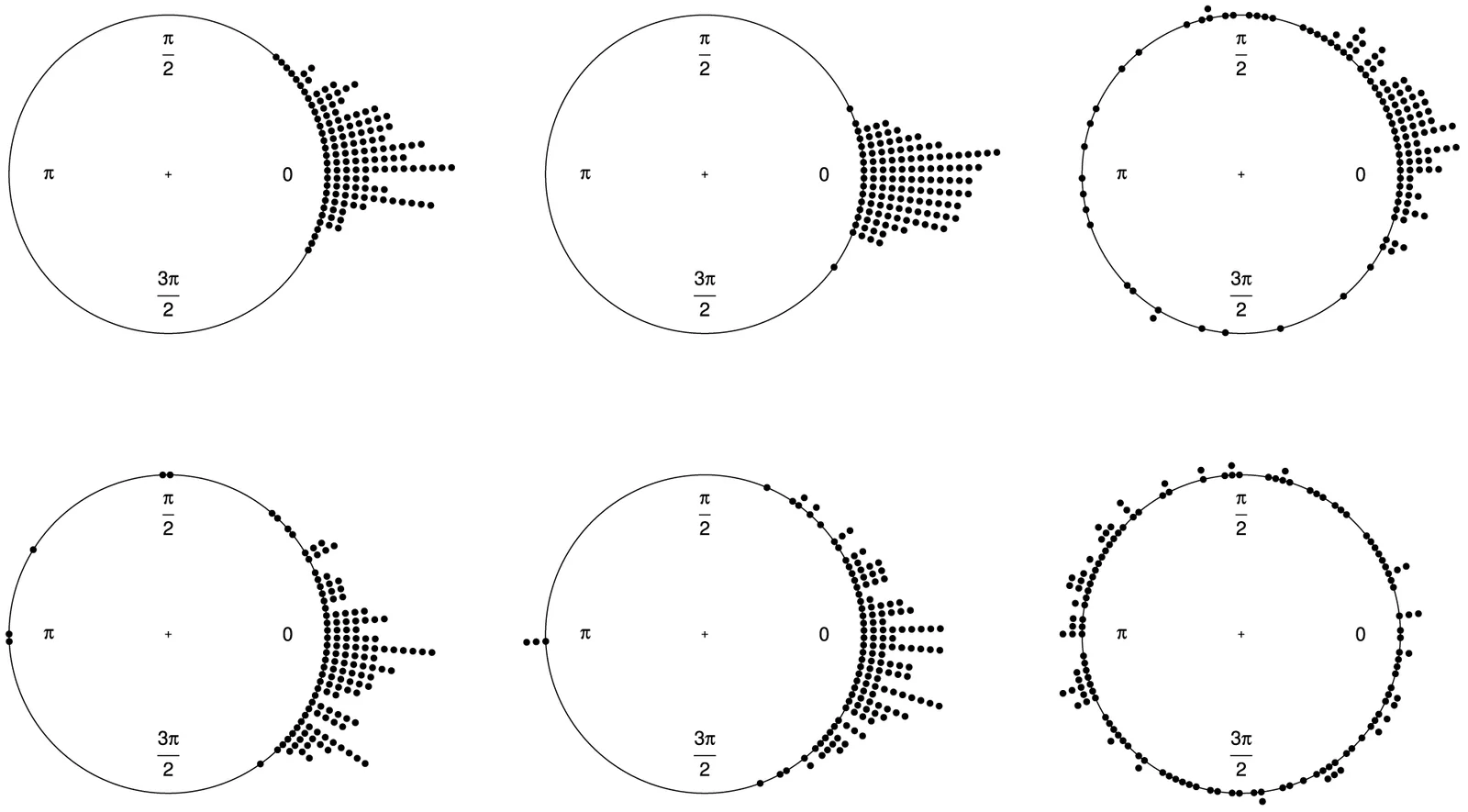
Circular plots showing the distribution of asynchronies as angles between partners **(left, center)** or between participants and the noisy metronome **(right)**. The left plots show data from the baseline, and the center and right plots show data from the same noisy metronome condition. The top plots are for a dyad who synchronized with each other with higher precision, and the bottom plots are for a dyad who synchronized with lower precision. In-phase relationships occur at 0, and anti-phase relationships occur at π.

### 3.2 Distractor influence

We predicted that the distractor would influence participants' timing, especially in the LRC, perfect fifth, and no-visual-contact conditions. Overall, cross-correlations were stronger between partners (*M* = 0.37) than between participants and the noisy metronome (*M* = 0.08), *t*_(549)_ = 29.4, *p* < 0.001, and *d* = 1.25, which means that partners were more influenced by each other than they were by the noisy metronome. We found no interaction between distractor noise and comparison, no main effect of distractor noise, and no covariate effects on correlation coefficients, but there was a main effect of comparison, *F*_(1, 235)_ = 563.9, *p* < 0.001, and $\eta_{p}^{2}$ = 0.71, showing again that partners were influenced more by each other than by the metronome. Likewise, we found no interaction between distractor pitch and comparison, no main effect of distractor pitch, and no covariate effects on correlation coefficients, but there was again an effect of comparison, *F*_(1, 141)_ = 508.6, *p* < 0.001, and $\eta_{p}^{2}$ = 0.78.

An interaction arose between visual contact and comparison, *F*_(1, 141)_ = 9.63, *p* = 0.002, and $\eta_{p}^{2}$ = 0.06, with no main effect of visual contact and no covariate effects, but a main effect of comparison, *F*_(1, 141)_ = 519.70, *p* < 0.001, and $\eta_{p}^{2}$ = 0.79. *Post-hoc* tests with a significance threshold of α = 0.025, following Bonferroni correction, showed that visual contact marginally strengthened correlations between partners, *t*_(141)_ = 2.86, *p* = 0.025, and *d* = 0.52, but did not affect correlations between participants and the metronome, (*p*>0.025). Boxplots for distractor influence are shown in the middle line of Figure 3.

### 3.3 Tempo accuracy

We did not have any specific predictions about tempo accuracy, but examined it anyway to determine how successfully participants carried out the task instructions, which were to maintain the prescribed tempo while synchronizing with each other. Tempo was maintained more accurately with the noisy metronome (*M* = −4.95 × 10^−5^, *SD* = 2.82 × 10^−4^) than in the baseline (*M* = −4.00 × 10^−4^, *SD* = 5.64 × 10^−4^), *F*_(1, 23)_ = 9.79, *p* = 0.005, and $\eta_{p}^{2}$ = 0.30, with no significant covariate effects. The negative tempo accuracy values indicate a decreasing trend in IBIs over time; in other words, a tendency to speed up. When baseline data were excluded, there were no effects of distractor noise, distractor pitch, or visual contact, and no covariate effects for any of these models. Boxplots for tempo accuracy are shown in the bottom line of Figure 3.

We carried out some follow-up analyses to explore possible explanations for why the noisy metronome—which participants had been instructed to ignore—improved tempo accuracy. Since the baseline was always performed first, we supposed that the improvement in tempo accuracy might be attributable to practice. To test this, we carried out a set of paired t-tests comparing between the baseline and two first experimental trials. If tempo accuracy improved with practice, then we might see incremental improvement across these first three trials. Tempo accuracy was worse in the baseline than in trial 1, *t*_(19)_ = 2.93, *p* = 0.009, and *d* = 0.78, and trial 2, *t*_(19)_ = 3.90, *p* < 0.001, and *d* = 1.00, but did not differ between trials 1 and 2, *t*_(19)_ = 1.24, *p* = 0.23, and *d* = 0.39. These results suggest that the improvement in tempo accuracy was attributable to the presence of the noisy metronome rather than practice at drumming. A plot showing tempo accuracy per trial number is shown in Figure 5.

**Figure 5:**
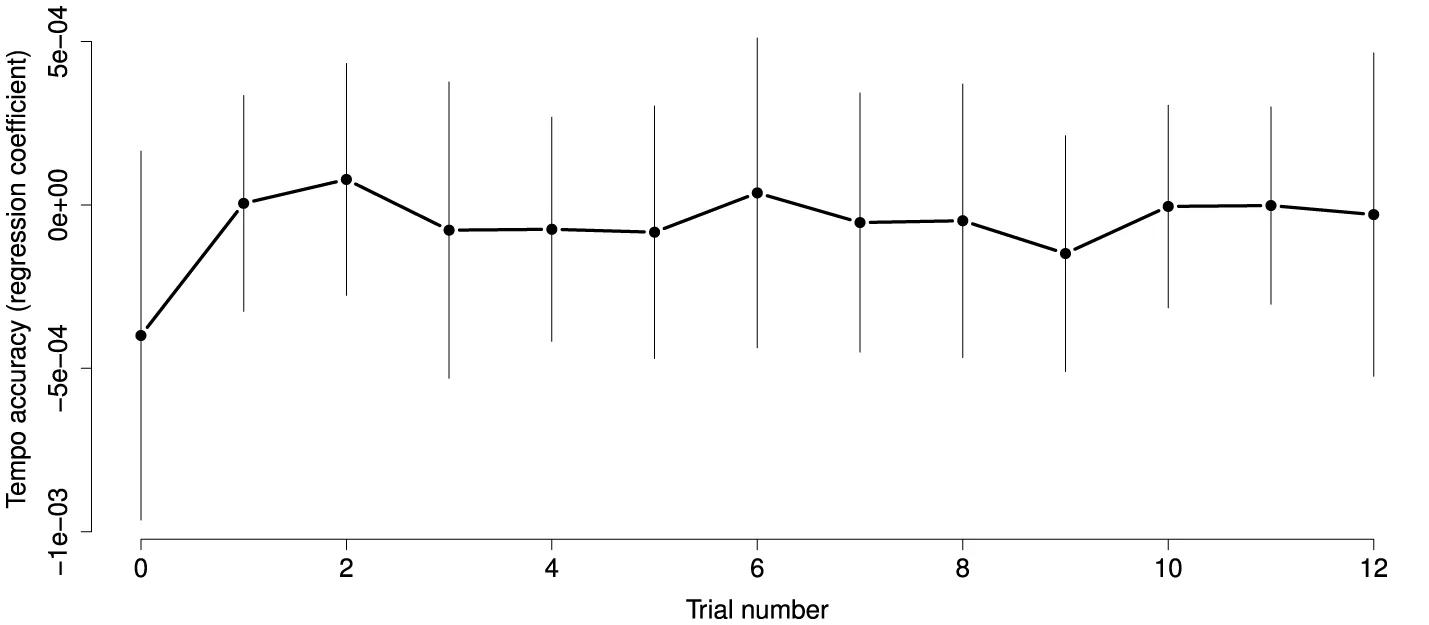
Average tempo accuracy per trial number. The baseline is coded as trial 0. The vertical lines at each value indicate standard deviation. A *y*-value of 0 would indicate perfect tempo accuracy.

Research by (Wohlschläger and Koch 2000) suggests that subdividing interbeat intervals might reduce rushing in sensorimotor synchronization tasks. Drawing on this, we supposed that the subdivision of participants' IBIs by metronome beats might explain the facilitatory effect of the noisy metronome on tempo accuracy. We ran a follow-up analysis to compare the duration of participant IBIs where subdivision occurred with the duration of participant IBIs where no subdivision occurred. Median IBI durations for subdivided and non-subdivided intervals were computed per trial. A paired *t*-test showed that subdivided intervals (*M* = 735.4) were slightly longer than non-subdivided intervals (*M* = 728.3), *t*_(566)_ = 4.60, *p* < 0.001, and *d* = 0.11. Thus, subdivision might have prompted lengthening of IBIs.

### 3.4 Quantity of head and vertical hand motion

We predicted that participants would move their heads and hands more in the presence of the noisy metronome, especially in the LRC and perfect fifth conditions, and that they would move more with visual contact than without. Our analysis showed more head motion with the noisy metronome than in the baseline, *F*_(1, 46)_ = 25.72, *p* < 0.001, $\eta_{p}^{2}$ = 0.36. Participant musical activity also had an effect, with active musicians moving more than inactive/nonmusicians, *F*_(1, 45)_ = 7.42, *p* = 0.009, and $\eta_{p}^{2}$ = 0.14; partner familiarity was nonsignificant. When baseline data were excluded, there were no effects of distractor noise, pitch, visual contact, or partner familiarity on head motion, though the covariate effect of musical activity remained in all cases, *F*_(1, 45)_ = 6.10, *p* = 0.02, and $\eta_{p}^{2}$ = 0.12 (model with distractor noise), *F*_(1, 45)_ = 5.93, *p* = 0.02, and $\eta_{p}^{2}$ = 0.12 (model with distractor pitch), *F*_(1, 45)_ = 6.01, *p* = 0.02, $\eta_{p}^{2}$ = 0.12 (model with visual contact).

There was more vertical hand motion with the noisy metronome than in the baseline, *F*_(1, 45)_ = 24.69, *p* < 0.001, and $\eta_{p}^{2}$ = 0.35. The model also showed an effect of partner familiarity, with participants moving more if they knew their partner than if they did not, *F*_(1, 44)_ = 4.92, *p* = 0.03, and $\eta_{p}^{2}$ = 0.10. The effect of participant musical activity approached significance, with slightly more motion among active musicians than inactive/nonmusicians, *F*_(1, 44)_ = 3.78, *p* = 0.06, and $\eta_{p}^{2}$ = 0.08. When baseline data were excluded, there were no effects of distractor noise, pitch, or musical activity on hand motion, but the covariate effect of partner familiarity was significant in both cases, *F*_(1, 45)_ = 5.05, *p* = 0.03, and $\eta_{p}^{2}$ = 0.10 (model with distractor noise), *F*_(1, 45)_ = 5.40, *p* = 0.02, and $\eta_{p}^{2}$ = 0.11 (model with distractor pitch). Again, this showed that participants moved more if they knew their partner than if they did not. The model testing for effects of visual contact showed more hand motion with visual contact than without, *F*_(1, 45)_ = 5.15, *p* = 0.03, and $\eta_{p}^{2}$ = 0.12, and an effect of partner familiarity, *F*_(1, 45)_ = 5.16, *p* = 0.03, and $\eta_{p}^{2}$ = 0.10. Boxplots for head and hand motion are shown in Figure 6.

**Figure 6:**
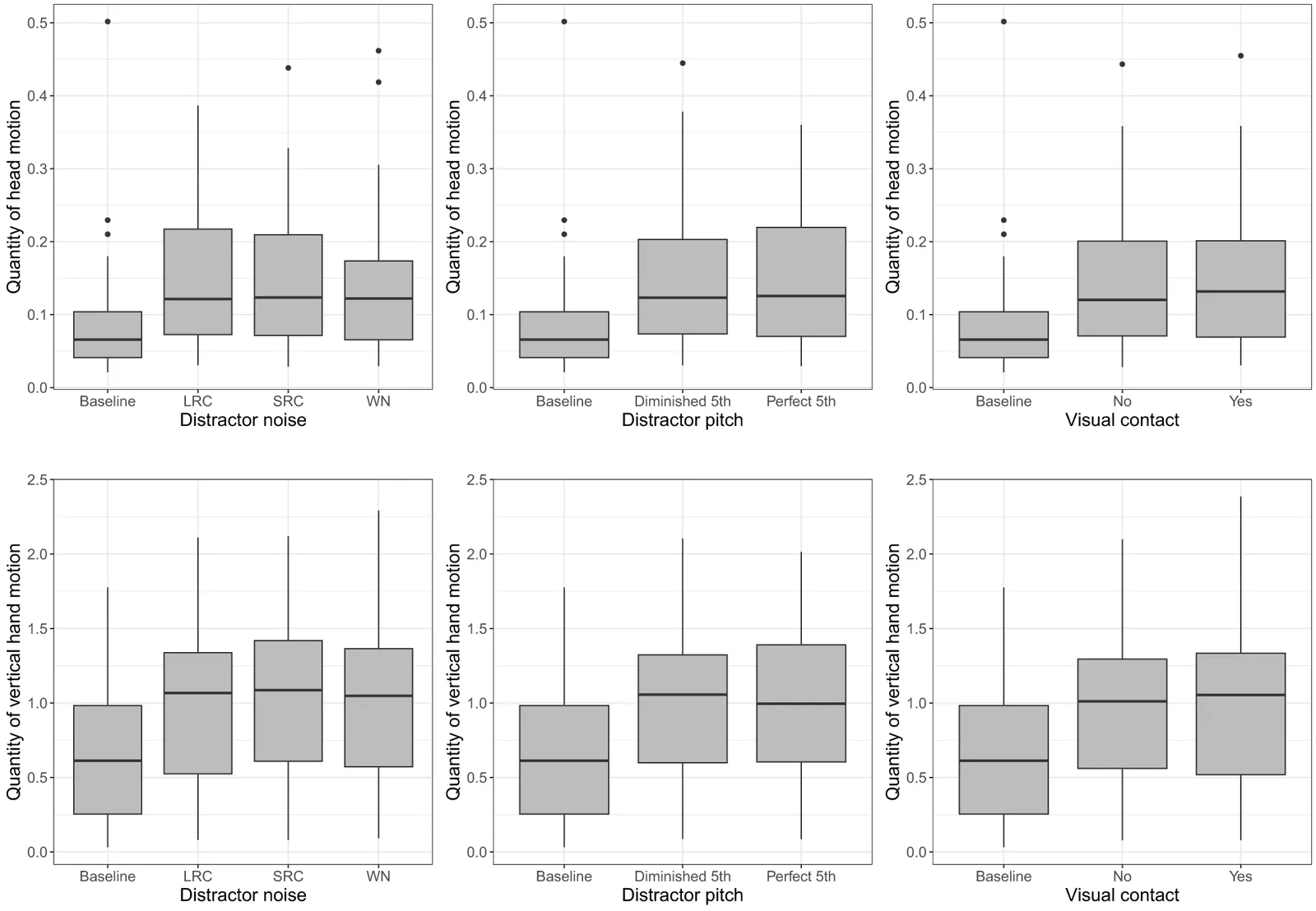
Effects of distractor noise, distractor pitch, and visual contact on quantity of head **(top)** and vertical hand motion **(bottom)**.

## 4 Discussion

This study investigated how a “noisy metronome” (i.e., a variable distractor rhythm) affects the stability of synchronization between people who are drumming an isochronous rhythm together. We manipulated the temporal and pitch characteristics of the noisy metronome and whether participants had visual contact while drumming. As expected, participants synchronized less precisely in conditions with the noisy metronome than in a baseline condition with no noisy metronome. However, they kept the prescribed tempo more accurately with the noisy metronome than without. The noisy metronome therefore destabilized synchronization while facilitating tempo-keeping. Synchronization precision, distractor influence, and tempo accuracy were all unaffected by manipulations of the noisy metronome's noise type and pitch. The presence of the noisy metronome prompted increased head and hand motion (measured in terms of speed), and hand motion was further increased when visual contact was permitted. Head and hand motion was also influenced by whether partners knew each other, and head motion was greater among active musicians. Visual contact improved synchronization between partners without affecting the correlation between participants and the noisy metronome. We discuss each of these findings below.

The noisy metronome destabilized synchronization between partners, causing reduced precision. Despite this, correlations between partners remained higher than correlations between individual participants and the noisy metronome. Thus, partners generally maintained weakened coordination with each other and resisted following the noisy metronome. Our results are in line with previous studies showing impaired synchronization in the presence of an acoustic distractor (Repp, 2003, 2004; Repp and Penel, 2004; Dalla Bella et al., 2013), but build on these studies by showing how distractor effects unfold in a live dyadic setting, in which participants act as each other's “target rhythm” and are mutually adaptive: We found that the noisy metronome was disruptive, but participants could still maintain a “loose” degree of synchrony through the two-minute trials.

We found no evidence to suggest that manipulations of distractor noise or pitch affected participants' abilities to maintain synchrony with their partner. Previously, Dalla Bella et al. (2013) found that music distractors perturbed participants' synchronization with a metronome more than did speech fragments, though the difference disappeared when speech and music shared the same meter. Thus, the disruptive power of a distractor seems to depend on the coherence and predictability of its temporal structure as well as the phase relationships between the distractor and target events. Our manipulations of noise—though they created rhythms that differed in structure and predictability—were likely too subtle to have differing effects on participants' attention.

The noisy metronome facilitated tempo-keeping, reducing the rushing that we observed during the baseline. Joint rushing is robustly observed in the rhythmic activity performed jointly by two or more people and is more pronounced in the activity performed by larger groups (Wolf et al., 2023). It results from a phase advancement mechanism that triggers a shortening of the subsequent period when a partner's action occurs during a critical time window before one's own action (Wolf et al., 2019). It was not surprising to see in the current study, since the tempo we chose was slower than the spontaneous motor tempo of most adults. Research suggests that the joint rushing effect is reduced when people perform rhythms that are more complex than sequences of isochronous beats. Interbeat intervals in more complex rhythms are subdivided, which reduces the variability of asynchronies and the likelihood that events fall into the critical period that triggers the phase advancement mechanism (Wolf et al., 2023). Subdivision of intervals also seems to affect the perception of interval duration (Repp, 2010). (Wohlschläger and Koch 2000) found that a rapid acoustic sequence presented at random times between metronome clicks prompted elimination of participants' tendencies to tap slightly in advance of the metronome (i.e., the negative mean asynchrony). Our experiment was similar in that the noisy metronome did not subdivide interbeat intervals at a fixed or integer ratio, given its variability and that of the participants. The novelty of our finding is that it shows the effect of interval subdivision on tempo perception can be caused by a rhythm that participants are deliberately ignoring. The ignored rhythm's effect on tempo-keeping is, therefore, automatic, and would require cognitive effort to resist.

We chose to use as a baseline a condition where partners synchronized without any metronome. Another possibility would have been to use a baseline where partners synchronized in the presence of a non-noisy metronome. With the latter, we would have been able to test whether the disruptions to synchronization stability that we observed with the noisy metronome were due to its noise or simply the presence of a third (unattended) rhythm. Ensemble musicians commonly use a shared metronome to improve their temporal coordination during rehearsals, but they prioritize attention to the metronome, in contrast to the paradigm that we used. To our knowledge, prior studies have not investigated the effect of an unattended non-noisy rhythm on dyadic synchronization.

Participants in our study engaged in various strategies for time-keeping during conditions with the noisy metronome. They moved more while hearing the noisy metronome than during the baseline, regardless of whether they could see their partner. This suggests that increased movement speed was important for regulating their own timing. Previous studies have shown that people make greater use of kinaesthetic cues, when regulating their timing is difficult, for example, by moving more forcefully if they are deprived of auditory feedback (Keller et al., 2010). However, most studies of body motion in interactive contexts focus on capturing the effects of interactivity, and do not account for changes that might also attributable to self-regulation. Our study showed that active musicians moved their heads more than inactive/nonmusicians in general, but we did not observe any interaction between musical activity and the noisy metronome, suggesting that this strategy of moving more was not contingent on musical experience.

We observed an additional effect of visual contact on drumming hand motion, which was further exaggerated when participants could see each other. This is in line with research showing that people move more when they are aware of being observed by a co-performer or audience (Bishop et al., 2019; Zimmermann et al., 2022). These studies investigated the expressively-complex cases of classical duo performance (Bishop et al., 2019) and co-improvised motion (Zimmermann et al., 2022); in contrast, our findings show that the effects of “being observed” also manifest during the minimally-expressive task of drumming a steady beat.

Participants benefited from being able to see their partner when the conditions allowed for visual contact, although they did not require visual contact to maintain an understanding of who was responsible for which pitch (Marozeau et al., 2010). This finding adds to a mixed literature that has shown the benefits of visual contact on the synchronization in some settings, but not in others (e.g., Bishop and Goebl, 2015; Colley et al., 2018). In our study, participants had a view of their partner's face and upper body, including the arm, hand, and drumstick as it struck the drum. Visual motion cues might have helped participants anchor their attention on their partner's rhythm, thereby facilitating auditory streaming. Anecdotally, many participants used other effectors to help with time-keeping—they nodded their head and/or tapped their foot in time with their drumming. Some also subdivided their drumming intervals by performing a mid-interval silent drumstrike. All of these strategies would have provided partners with additional motion cues as well as potentially facilitating self-regulation.

Maintaining synchrony with one of multiple concurrent auditory streams is a familiar task for ensemble musicians. Our participants ranged from non-musicians with no prior experience learning an instrument to highly trained semi-professional musicians. We did not control how dyads were constructed with regard to musical background, so some comprised two musicians, some two non-musicians, and some a combination of musician and non-musician. This unsystematic pairing can be considered a limitation of the study and might explain why we did not observe effects of musical activity on synchronization precision or distractor influence—participants' synchronization was not fully under their own control, since they had to cope with each other's variability. We did observe more head motion among active musicians than among inactive/nonmusicians, suggesting greater use of ancillary motion. This might reflect musicians' practiced strategies for timekeeping (see Laroche et al., 2022), communication (see Bishop et al., 2019), and/or heightened confidence and engagement with the drumming task. Musical activity had only a marginal effect on quantity of hand motion, which was increased more substantially by partner familiarity. This adds to previous literature showing that dyadic interaction is affected by partner familiarity, though previous studies have tended to focus on music- or movement-based coordination measures rather than quantity of motion (Latif et al., 2014; Golvet et al., 2021). Future research should follow up on our findings with more systematic tests of how musical activity and partner familiarity affect processes of auditory attention, synchronization, and time-keeping.

In conclusion, the central contribution of our study is a demonstration of how distractor rhythms with a simple timing structure can perturb synchronization while simultaneously helping participants maintain their tempo. Our findings suggest that the processing of interval durations during a synchronization task—and the effects of subdivision on that processing—occurs partly automatically. Also notable is how participants made use of their bodies to support their performance on the task, drawing on movement cues both for communication with their partner and for their own self-regulation. The task that we used is a simplified paradigm compared to what ensemble musicians do, which can involve attending selectively to one of many different concurrent voices, each presenting their own variability on top of a complex musical structure. Future research should investigate how different structural elements, such as expressive timing variability and pitch contour, influence players' abilities to maintain their attention on a selected voice.

## Data Availability

The datasets generated for this study can be found here: https://zenodo.org/records/13376172.
